# Impact of Fecal Calprotectin Measurement on Decision-making in Children with Inflammatory Bowel Disease

**DOI:** 10.3389/fped.2017.00007

**Published:** 2017-01-25

**Authors:** Wael El-Matary, Esmail Abej, Vini Deora, Harminder Singh, Charles N. Bernstein

**Affiliations:** ^1^Department of Pediatrics and Child Health, Section of Pediatric Gastroenterology, University of Manitoba, Winnipeg, MB, Canada; ^2^The University of Manitoba IBD Clinical and Research Centre, Winnipeg, MB, Canada; ^3^The Children’s Hospital Research Institute of Manitoba, Winnipeg, MB, Canada; ^4^Department of Internal Medicine, Section of Gastroenterology, University of Manitoba, Winnipeg, MB, Canada

**Keywords:** calprotectin, children, colitis, Crohn, IBD

## Abstract

**Background:**

The use of fecal calprotectin (FCal) as a marker of intestinal inflammation, in the management of inflammatory bowel disease (IBD) is increasing. The aim of this study was to examine the impact of FCal measurements on decision-making and clinical care of children with IBD.

**Materials and methods:**

In a retrospective cohort study, FCal, clinical activity indices, and blood markers were measured in children with established diagnoses of IBD. Pearson correlation coefficient analysis was performed to examine association between FCal and other markers. Decisions based on FCal measurements were prospectively documented and participants were evaluated 3–6 months later.

**Results:**

A total of 115 fecal samples were collected from 77 children with IBD [median age 14, interquartile range (IQR) 11–15.6 years, 42 females, 37 with Crohn’s disease]. FCal positively correlated with clinical activity indices (*r* = 0.481, *P* < 0.05) and erythrocyte sedimentation rate (*r* = 0.40, *P* < 0.05) and negatively correlated with hemoglobin (*r* = −0.40, *P* < 0.05). Sixty four out of 74 (86%) positive FCal measurements (≥250 μg/g of stools) resulted in treatment escalation with subsequent significant clinical improvement while in the FCal negative group, 34 out of 41 (83%) measurements resulted in no change in treatment and were associated with remission on follow-up.

**Conclusion:**

Based on high FCal, the majority of children had treatment escalation that resulted in clinical improvement. FCal measurements were useful and reliable in decision-making and clinical care of children with IBD.

## Introduction

Stool markers such as fecal calprotectin (FCal) have emerged as new diagnostic tools to help in the diagnosis of intestinal inflammation (1). Fecal inflammatory markers include a biologically heterogeneous group of substances that either leak from or are actively released by the inflamed mucosa. Calprotectin is a small calcium binding protein consisting of two heavy and one light polypeptide chains (a heterodimer of two S100 proteins) (1). It is found in neutrophilic granulocytes, accounting for 60% of their cytosolic fraction, and in monocytes and macrophages (1–3). It has a homogeneous distribution in feces. In active inflammatory bowel disease (IBD), an increased migration of inflammatory cells including neutrophils to the inflamed intestinal mucosa has been observed. Due to leukocyte shedding in the intestinal lumen, pro-inflammatory proteins such as calprotectin can be detected and measured in the stools. FCal demonstrates good sensitivity and specificity as a marker for mucosal inflammation (1–4).

Inflammatory bowel disease is a chronic disease characterized by remissions and relapses and encompasses two related but distinct disorders of as yet unknown etiology: Crohn’s disease (CD) and ulcerative colitis (UC) (5). Both adult and pediatric investigators have recognized the need to optimize and standardize methodology for assessment of disease activity. In children, active disease can have devastating consequences on growth and development. Consequently, it is prudent that disease remission is induced and maintained (6). The goal for IBD therapy is increasingly becoming mucosal healing. Therefore, the need for non-invasive, reliable, and relatively non-expensive biomarkers for IBD disease activity is growing (7).

Fecal calprotectin concentrations may correlate well with both endoscopic and histological IBD activity, which have been traditionally used to evaluate mucosal healing and response to treatment (8–10). However, the impacts of FCal measurement on decision-making and clinical outcomes in children with IBD are currently under-reported. The aim of this study was to examine the impact of FCal measurements on decision-making and clinical care of children with IBD in usual clinical care.

## Materials and Methods

### Study Population

In a single centre cohort study, consecutive children with established diagnoses of IBD assessed between November 2013 and December 2015 in the Pediatric IBD Outpatient Clinic at the Children’s Hospital, Winnipeg, MB, Canada, who had developed new symptoms that might had suggested a disease relapse were asked to bring in a stool sample for FCal measurement. Inclusion criteria included all children (<18 years) with confirmed diagnosis of IBD following the North America Society of Pediatric Gastroenterology, Hepatology and Nutrition diagnostic criteria for IBD (11).

### Covariate Data

Patients’ demographics and disease characteristics and duration were collected from patients’ medical records. The most significant symptom or sign as an indication for requesting FCal measurement was prospectively documented for each collected sample.

#### Fecal Calprotectin

Fecal samples were collected at home and processed at the laboratory of the University of Manitoba IBD Clinical and Research Centre. FCal measurement was performed using the Quantum Blue^®^ Lateral Flow Reader within 24 h of stool collection. FCal ≥ 250 μg/g of stools was considered a positive test, indicative of active IBD. The upper limit of test was >1,800 μg/g of stools.

#### Clinical Activity Indices

Clinical activity indices [pediatric UC activity index (PUCAI) for UC and pediatric Crohn’s activity index (PCDAI) for CD] (12, 13) and the impact of FCal testing on the management of IBD—investigations or changes in therapy—were assessed. Physician global assessment (PGA) for disease activity, whether it is quiescent, mild, moderate, or severe, was also documented (14).

#### Other Laboratory Markers

Included hemoglobin, erythrocyte sedimentation rate (ESR), C-reactive protein (CRP), and serum albumin performed within 2 weeks of stool collection for FCal.

When patients were asked to bring stool samples, pediatric gastroenterologists were asked to complete a questionnaire. Other investigations such as hemoglobin, serum albumin, and CRP were known to physicians who made no changes in treatment at that time point. All patients with diarrhea had their stools tested for infection screen and only those with negative infection screen had stools for FCal measurements. The clinicians were asked to decide if they would do any additional investigations (mainly colonoscopy) or any changes in therapy, based only on the FCal results. Clinicians were given specific options to choose from for treatment escalation in case FCal comes back as positive. Once the FCal results were known, any investigations and treatment changes were documented and patients were followed up for 3–6 months. Clinical activity indices were measured in the follow-up visits.

If colonoscopy was performed following FCal results, colonoscopy findings and histopathology of collected mucosal biopsy samples performed within 1 month of collecting stool samples were examined. Presence of erythema, loss of vascularity, friability, and/or ulcerations were included in the definition of active disease on endoscopy (active vs. inactive).

### Statistical Analysis

Calculations and data analysis were performed using Statistical Package for the Social Sciences (SPSS, IBM Corp., 2013, IBM SPSS Statistics for Windows, Version 22.0. Armonk, NY, USA). Descriptive measures [medians, interquartile range (IQR), means, ranges, and SD] were calculated for continuous variables (such as age and clinical disease activity indices), while frequencies were calculated for categorical variables (such as PGA), along with 95% confidence intervals (CIs) for the means and proportions. Variables were examined for normal distribution. Student’s *t*-test was used to compare means. Categorical variable comparisons were performed using Fisher’s exact test. Pearson correlation coefficient analysis was performed to examine any possible association between FCal values and clinical disease activity indices, ESR, CRP, serum albumin, hemoglobin (all continuous variables), PGA (as a categorical variable; quiescent, mild, moderate, and severe), and endoscopic activity (as a dichotomous variable; active vs. inactive). A *P* value < 0.05 was considered significant.

### Ethical Considerations

The study protocol was approved by the University of Manitoba Health Research Ethics Committee. As the study was retrospective, individual consents from participants were not needed.

## Results

Over the study period, a total of 115 fecal samples were collected from 77 children with IBD (median age at FCal measurements 14, IQR 11–15.6 years, 42 females, 37 with CD), who were followed up for a median duration of 1.33 (IQR 0.5–3.1) years (mean 2.62 ± 1.84 years). Participants’ demographics and disease distribution are summarized in Table 1. Base line medications were summarized in Table 2.

**Table 1:** **Demographic characteristics and disease phenotype in participants**.

Number	77

**Gender**	
Male	35
Female	42

**Diagnosis**	
Ulcerative colitis (UC)	37
Crohn’s disease (CD)	38
IBD-U	2

**Age**	
Median	14
Mean	13.08
Range	3.16–17.25

**UC phenotype**	
E1	3
E2	5
E3	16
E4	15

**CD phenotype**	
**Location**	
Small bowel	7
Colon	8
SB-Colon	23
Upper GI^a^	19
Perianal	12

**Behavior**	
Inflammatory	29
Stricturing	7
Fistulizing	2

*^a^Upper GI involvement in combination with other phenotypes*.

**Table 2:** **Base line medications of all participants**.

Medication	Number^a^
5-ASA	55
Azathioprine	38
Methotrexate	11
Infliximab	31
Adalimumab	9
Enteral nutrition	3
Steroids	11
Antibiotics	1

*^a^Several patients were on more than one medication*.

The most significant symptom or sign such as diarrhea, weight loss, and pallor as an indication for FCal measurement is listed in Table 3.

**Table 3:** **Indications (most significant symptom/sample) for fecal calprotectin measurements in 77 children with inflammatory bowel disease**.

Abdominal pain	38
Bleeding per rectum	24
Diarrhea	22
Weight loss	15
Fatigue	8
Others (e.g., constipation, abnormal labs)	8

Total	115

Seventy four (64%) stool samples were positive (≥250 μg/g) and 12 participants had colonoscopy within 4 weeks of FCal measurement as compared with two patients in the group with normal FCal measurements (*P* < 0.001).

### Association between FCal and Study Co-Variables

For the whole cohort, 74 (66%) out of 115 fecal samples had high FCal > 250 μg/g of stools. In 33 (44.6%) out 74 positive fecal samples, clinical activity indices were ≥10. On the other hand, 41 (34%) out 115 fecal samples had normal FCal level and only 2 (5%) out of 41 samples had clinical activity indices ≥10. Both patients were scoped and colonoscopy was normal.

Fecal calprotectin measurements had fair correlation with clinical disease activity indices (*r* = 0.483, *P* < 0.05), PGA (*r* = 0.40, *P* < 0.05), ESR (*r* = 0.40, *P* < 0.05), low hemoglobin (*r* = −0.40, *P* < *0.05*), with less correlation with low serum albumin (*r* = −0.3, *P* < 0.05) but no correlation with CRP (*r* = 0.1, *P* = 0.3).

When those with CD were compared to the UC/IBD-U group, the correlation of FCal with clinical disease activity indices was stronger for UC compared to CD. High FCal levels correlated with PUCAI (*r* = 0.80, *P* < 0.01) compared to PCDAI (*r* = 0.2, *P* = 0.09).

Correlation was limited (*r* = 0.256, *P* = 0.006) with endoscopic activity as only 14 children had colonoscopy within 4 weeks of FCal measurement.

### Impact on Management

Only 14 (12%) out of 115 samples resulted in colonoscopy as guided by physicians’ discretion (likely when physicians felt that FCal value was equivocal between 100 and 250); 2 in FCal negative and 12 in the FCal positive group, i.e., for the rest of the cohort [101 (88%) samples], decisions on treatment (escalation or no treatment) were based solely on FCal measurements. Sixty four out of 74 (86%) samples with positive FCal measurements were associated with treatment escalation that resulted in improvements in clinical activity indices (Table 4), while in the FCal negative group, 34 out of 41 (83%) measurements were associated with no change in treatment.

**Table 4:** **Changes in treatment based on 74 fecal samples with abnormally high fecal calprotectin**.

Change in medications	Change in medications (%)	Mean disease activity index before the change	Mean disease activity index after the change	*P* value, 95% confidence interval
Starting/switching a biologic	18 (24.3%)	20 ± 15.86	5 ± 2.04	*P* < 0.05, 6.81–14.43
Increase the dose/reduce time interval of a biologic	15 (20.3%)	16.6 ± 9.14	12.03 ± 8.37	N.S, −2.28 to 12.28
Increase dose of the immunomodulator	15 (20.3%)	13.8 ± 7.61	8.63 ± 6.3	N.S, −0.67 to 13.4
Adding an immunomodulator	5 (6.6%)	23 ± 8.3	6.25 ± 4.33	*P* < 0.05, 3.25–26.71
Adding steroids	7 (9.4%)	17.14 ± 9.49	13.12 ± 9.43	N.S, −18.14 to 11.84
Increase/add 5-ASA	7 (9.4%)	15.63 ± 10.48	7.5 ± 6.123	N.S, −6.17 to 22.44
No change of treatment	10 (2 declined change) (13.5%)	10.83 ± 6.05	5.7 ± 4.3	N.S, −5.8 to 18.4
Total	67^a^	12.83 ± 12.68	5.25 ± 7.15	*P* < 0.05, 4.48–11.34

*^a^Sixty-four changes in treatment (three samples resulted in more than one change in medication)*.

**Figure 1:**
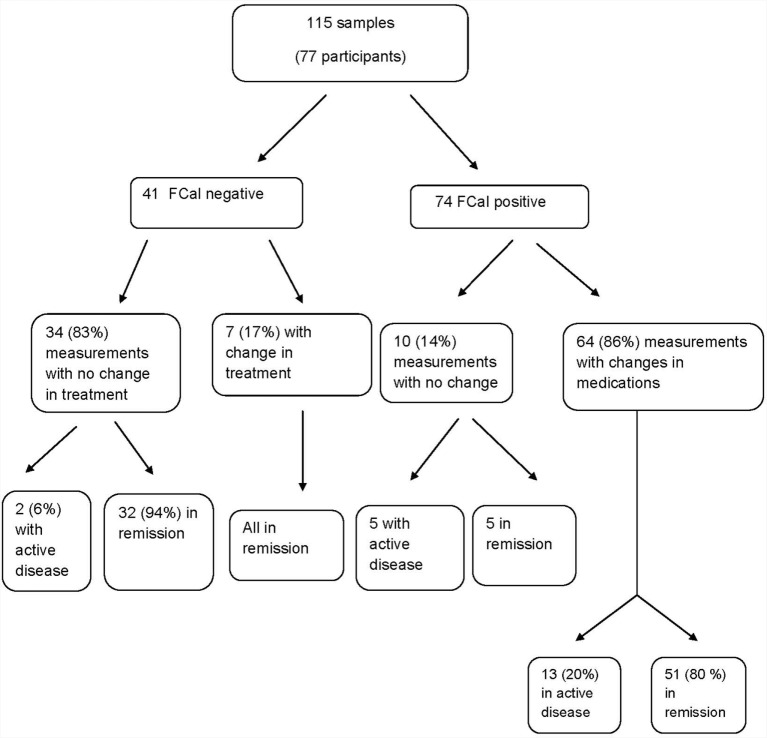
**Flow chart summarizing the impact of fecal calprotectin measurements on the decision-making process among children with inflammatory bowel disease**.

Figure 1 summarizes the impact of FCal measurements on the study cohort over time. In the first follow-up visit 3–6 months following changes in treatment in the FCal positive group, of those measurements that resulted in treatment escalation, 80% ultimately achieved clinical remission while in the FCal negative group, 32 out 34 (94%) of those who did not have any treatment changes (including 2 patients with clinical activity indices >10), were in clinical remission at the follow-up visit.

## Discussion

Fecal calprotectin is a surrogate marker for mucosal inflammation and has been proven as a very beneficial, non-invasive, and relatively an inexpensive test in the work-up of IBD with a considerable high sensitivity (15, 16). Although high FCal can be found in other gastrointestinal disorders, in addition to IBD, such as gastrointestinal infections and polyposis, proper use of this test may reduce the need for frequent colonoscopies (15).

Our study showed a fair positive correlation of high FCal with increased clinical disease activity, a finding that is concordant with other adult and pediatric studies (9, 17–19). It is noteworthy that 55% of persons with increased FCal were in clinical remission by a symptom based activity index. While this may lead some to consider that this suggests that either the FCal is non-specific, or that a substantial proportion of children with IBD in remission have active inflammatory disease. The majority of our subjects with elevated FCal had treatment escalation (even if disease activity index suggested remission). In pediatric IBD practice, our study is unique in that we relied on FCal to direct clinical management and then followed up to determine if our FCal-directed management led to satisfactory clinical outcomes several months later.

Bunn et al. examined FCal in spot fecal samples from 37 children with IBD and 31 control children. FCal was higher in 16 children with UC and in 21 children with CD (18). Similar to our study, FCal concentration correlated negatively with serum albumin concentration (18). In another study from Italy, FCal showed a high correlation with the histologic grade of mucosal inflammation, similar to that observed for endoscopy in 58 children with IBD (19).

In our study, FCal, did not correlate that well with CRP, a finding which was also highlighted in other studies (20, 21). Schoepfer and colleagues concluded that FCal correlated better with endoscopic scoring than that with CRP (20, 21). Correlations with CRP, however, were *r* = 0.53 and 0.55 in 140 ileocolonic CD and 228 UC adult participants, respectively (18, 19). In our study, the assessment of association between high FCal and endoscopic disease activity was limited by the small number of patients who had colonoscopy and the non-validated categorical classification of endoscopic activity into two subcategories (active and inactive disease).

Our study showed a better correlation of FCal with PUCAI compared to PCDAI. FCal has been previously also found to be a stronger marker for UC compared to CD (22).

Monitoring disease activity in patients with IBD is crucial as mucosal healing has become now a new target for treatment. It has been established that calprotectin concentrations correlate well with endoscopic and histological IBD activity (8, 9). However, data on the impact of FCal directing management in an IBD population, especially in children, are sparce. In our study, 86% of positive samples resulted in escalation of treatment with subsequent improvement in the majority of participants and 88% of negative samples resulted in no change of treatment and sustained clinical remission in the majority of participants in subsequent follow-up.

In a recent study from The Netherlands, clinical activity indices, FCal, and CRP were measured in 62 teenagers with IBD (31 with CD and 31 with UC), who were asked to evaluate their disease control (100% was the best disease control) and their disease control, according to participants’ opinion, was above 90% in two successive outpatient clinic visits. Unlike CRP, positive FCal was predictive of a clinical relapse in the subsequent visit in 60% of participants (23). In another prospective multicentre cohort study, stool samples from 101 children who were admitted with severe UC were obtained for measuring four different stool markers for inflammation including FCal. Repeat samples at discharge were obtained from 24 children. Although all markers were initially significantly elevated, FCal did not correlate that well with PUCAI which performed better than the fecal markers in predicting outcome following a course of intravenous corticosteroids. However, perhaps it was too soon to expect a significant drop in FCal following the short course of intravenous corticosteroids (24).

Eccles et al. collected data on the impact that FCal testing from 119 clinic participants including 29 with IBD. FCal testing helped in assessment of disease activity and decision-making in 25 adults with known IBD; 21 had major changes in their treatment while 4 were felt to be in remission on biological therapy (25). In four pediatric participants with known IBD, one had a normal FCal level and was felt to be in remission but three had elevated levels that resulted in major changes in management. Similar to our study, the investigators concluded that FCal can serve as a marker for disease activity in known IBD and help in the clinical decision-making process (25).

In an open label randomized controlled study, Osterman et al. observed significantly dropping FCal levels in adults with quiescent UC after escalating their mesalamine dose (26). In another randomized study, Lasson et al. used FCal measurements to identify persons with UC who were at risk of relapse and escalated their therapy (27). Both studies, however, were experimental and not reflective of routine real-life clinical practice.

The limitations of our study include the retrospective design and small sample size especially for those who had colonoscopy. However, based on previous research, FCal is known to strongly correlate with endoscopic disease activity (9, 17) and we assumed that it did. Hence, the escalation of treatment in most participants with elevated FCal levels regardless of whether a colonoscopy was performed or not. The study main aim was how FCal measurements, as the major decisive factor, would impact on disease management. A positive FCal most often triggered a clinical response; especially a change in therapy. As we did not do colonoscopy in all subjects we cannot discern how many false negative FCal measurements were represented within our sample. Nonetheless, our results did show that the faith clinicians placed in the FCal result was rewarded with good clinical outcomes. Another limitation was a lack of measurements of FCal in the follow-up visits after implementing changes in therapy but the study outcome was intended to be clinical activity indices. Nonetheless, the study adds significantly to the current literature as little is known about the impact and outcome of FCal measurements in pediatric IBD clinical practice.

## Conclusion

In children with known IBD, elevated levels of FCal were more likely to be associated with elevated scores of clinical activity indices, high ESR, and low hemoglobin. However, many children with elevated FCal had normal clinical disease activity indices. Regardless of disease activity index at the time of FCal, based on abnormal measurements of FCal, the majority of children had their treatment escalated which resulted in significant clinical improvement in those with elevated disease activity index or sustained remission in those in whom the index was considered in remission. On the other hand, the majority of those with normal FCal did not have any changes in their investigations or treatment and remained in clinical remission which saved patients and health-care resources from unnecessary interventions.

## Author Contributions

All authors made substantial contributions to the conception or design of the work; or the acquisition, analysis, or interpretation of data for the work; drafting the work or revising it critically for important intellectual content; final approval of the version to be published; and agreement to be accountable for all aspects of the work in ensuring that questions related to the accuracy or integrity of any part of the work are appropriately investigated and resolved.

## Conflict of Interest Statement

WE-M has served as an advisory board member for Janssen Canada and AbbVie Canada and received research support from Janssen Inc. HS has consulted to Medial Cancer Screening Ltd., Israel, served on advisory board for Pendopharm, and received research funding from Merck Canada. CB is supported in part by the Bingham Chair in Gastroenterology. He has served on advisory boards for AbbVie Canada, Ferring Canada, Janssen Canada, Shire Canada, Pfizer Canada, and Takeda Canada. He has consulted to Mylan Pharmaceuticals and Bristol Myers Squibb. He has received unrestricted educational grants from AbbVie Canada, Janssen Canada, Shire Canada, and Takeda Canada. He has been on speaker’s bureau for AbbVie Canada and Shire Canada. The other authors declare no conflict of interest.

## Abbreviations

FCal, fecal calprotectin; IBD, inflammatory bowel disease; CD, Crohn’s disease; UC, ulcerative colitis.
